# Editorial: Neural mechanisms of impulsivity and compulsivity and their clinical and therapeutic corollaries

**DOI:** 10.3389/fnbeh.2022.991267

**Published:** 2022-08-11

**Authors:** Charidimos Tzagarakis

**Affiliations:** ^1^Department of Neuroscience, University of Minnesota, Minneapolis, MN, United States; ^2^Organization Against Drugs (OKANA), Athens, Greece

**Keywords:** impulsivity, compulsivity, Ventral Striatum, Deep Brain Stimulation (DBS), Iowa Gambling Task (IGT), Nucleus Accumbens, global cerebral ischemia (GCI)

Anyone paying attention to the evolution of diagnostic (DSM-5, ICD-11) and research (RDoC- Insel et al., 2010) criteria for psychopathology in the past decade will observe an effort by clinicians and basic scientists alike to redefine our approach to psychopathology in a way that incorporates ever accumulating results from basic and clinical neuroscience and allows for novel and useful questions about diagnosis and treatment to be posed. In this process, the concepts of impulsivity and compulsivity are emerging as particularly relevant constructs (Robbins et al., 2012). The former tends to refer to styles of acting and making decisions with limited foresight and a proneness to errors (Tzagarakis et al., 2019), whilst the latter marks, in contrast, a behavioral “stickiness” with an abnormal perseveration on actions and strategies that are often maladaptive (Luigjes et al., 2019; Muela et al., 2022). Crucially, impulsivity and compulsivity can sometimes be perceived to mutually reinforce a vicious cycle in a number of conditions, such as gambling (Bowden-Jones et al., 2022) and, possibly, eating disorders, where both traits can co-occur (Howard et al., 2020). This observation and the questions it inevitably generates regarding possible shared or closely interrelated neurophysiological mechanisms (Belin et al., 2008) are at the core of this Research Topic. We were therefore happy to see submissions that were diverse in the methodology used and research paths pursued, but ultimately also highlighted striking physiological commonalities.

In two papers submitted in tandem (Scaife et al.; Braeutigam et al.), a group from the University of Oxford present results from a pioneering study of the treatment of severe anorexia nervosa using Deep Brain Stimulation (DBS) with electrodes implanted bilaterally in the Nucleus Accumbens. Only some of the seven participants in the study responded to DBS in what highlights future challenges that need to be addressed in order to define the place of this treatment modality in the management of severe eating disorders. In particular, factors such as electrode localization, prognostic patient profiles and their interaction will all need to be clarified in future work with larger cohorts. However, the study's design, including blind on vs. off DBS phases, detailed psychometrics and crucially, the longitudinal collection of neural data in the form of Magnetoencephalography (MEG) (Braeutigam et al.) sets a very useful reference point for the field and already offers tantalizing clues regarding the role of compulsivity in the pathophysiology of restrictive eating disorders, as there are indications of a correlation between response to treatment and pre-existing OCD. The use of MEG highlights the ever increasing usefulness of human electrophysiology in providing quality neural data that can drive hypothesis formation and testing in the realm of clinical research.

Using different techniques, but here too tackling a disease process where compulsive behavior is prominent. Hasuzawa et al. report on the resting state BOLD fluctuations (fractional amplitude of low frequency fluctuations—fALFFs) of a cohort of OCD patients vs. healthy controls, in relation to their performance in the Iowa Gambling Task, a neuropsychological test sensitive to pathological decision styles, where participants attempt to maximize long-term value accumulated by selecting cards from decks with different discounting profiles. Much like with the stimulated Accumbens in the anorexia studies, the Ventral Striatum seems to take center stage again, with higher putamen signal for the patient cohort compared to controls and a possible association to abnormally high sensitivity to Response Prediction Errors.

Finally, further adding to our insights into impulsivity/compulsivity, comes a non-human study of the effects of global cerebral ischemia on the neurochemistry and associated impulsive behavior of rats, by Morin et al. The Nucleus Accumbens is again implicated with Dopamine neurotransmission shown to be negatively affected. This does not however result in an increase in impulsive discounting, but rather has effects on the animals' motor behavior which may be associated with impulsive action. This dissociation of impulsive choice and impulsive action highlights the need to continue the exploration of the structure of the impulsivity and compulsivity constructs.

In their individual diversity and originality but also their striking commonalities, the papers in this Research Topic constitute significant contributions both to their specific respective fields and, at the same time, to the emerging understanding of the neurobiology and pathophysiology of the impulsivity/compulsivity dimension.

## Author contributions

The author confirms being the sole contributor of this work and has approved it for publication.

## Conflict of interest

The author declares that the research was conducted in the absence of any commercial or financial relationships that could be construed as a potential conflict of interest.

## Publisher's note

All claims expressed in this article are solely those of the authors and do not necessarily represent those of their affiliated organizations, or those of the publisher, the editors and the reviewers. Any product that may be evaluated in this article, or claim that may be made by its manufacturer, is not guaranteed or endorsed by the publisher.
